# Supplementary material for: Flexible Implementation of the Trilinearity Constraint in Multivariate Curve Resolution Alternating Least Squares (MCR-ALS) of Chromatographic and Other Type of Data
Source: Molecules. 2022 Apr 5;27(7):2338. doi: 10.3390/molecules27072338 (PMC9000239; doi:10.3390/molecules27072338)
Supplement: Supplementary file 1 [file molecules-27-02338-s001.zip › molecules-1639199-supplementary.pdf]

Table S1. Comparison of the multiple sample unfolded elution profiles obtained by ATLD, PARAFAC, PARAFAC2, MCR-ALS 1,1,0, MCR-ALS 2,2,0, MCR-ALS 1,1,1, MCR-ALS 0,0,0, with that of MCR-ALS 2,2,1 in the analysis of the wine dataset. See Equations 9-10 for the meaning of r^2 and angle. See end of section 2.2 for the meaning of the different MCR-ALS variants

		r^2	Angle
ATLD	3-hydroxy-2-butanone	0.9100	24.4
	hexyl acetate	0.9186	23.3
	Background	0.9996	1.6
PARAFAC	3-hydroxy-2-butanone	0.9364	20.5
	hexyl acetate	0.9116	24.2
	Background	0.9996	1.52
PARAFAC2	3-hydroxy-2-butanone	0.9884	8.8
	hexyl acetate	0.9914	7.5
	Background	0.9996	1.7
DNTD	3-hydroxy-2-butanone	0.9829	10.6
	hexyl acetate	0.9857	9.7
	Background	0.9996	1.7
MCR-ALS 1,1,0	3-hydroxy-2-butanone	0.9250	22.3
	hexyl acetate	0.9252	22.3
	Background	0.9680	14.5
MCR-ALS 2,2,0	3-hydroxy-2-butanone	1.0000	0.2
	hexyl acetate	1.0000	0.2
	Background	0.9713	13.7
MCR-ALS 1,1,1	3-hydroxy-2-butanone	0.9252	22.3
	hexyl acetate	0.9256	22.2
	Background	0.9999	0.9
MCR-ALS 0,0,0	3-hydroxy-2-butanone	0.9932	6.7
	hexyl acetate	0.9916	7.4
	Background	0.9729	13.4

Table S2. Comparison of results obtained by ATLD, PARAFAC, PARAFAC2, and different variants of MCR-ALS using 3 components in the analysis of the FIA dataset. In bold red worse recovered profiles with r^2 values below 0.9. See Equations 7-10 for the meaning of R^2, lof, r^2 and angle. See end of section 2.2 for the meaning of the different MCR-ALS variants

	R^2	lof	Recovery of sample profiles					
			2HBA		3HBA		4HBA	
			r^2	Angle	r^2	Angle	r^2	Angle
Three components model (one per chemical compound)								
ATLD	67.6	56.9	0.8633	30.3	0.9927	6.9	0.9181	23.4
PARAFAC	88.2	34.2	0.9382	20.2	**0.7137**	**44.5**	**0.7204**	**43.9**
PARAFAC2	99.9	0.8	0.8665	29.9	0.9679	14.6	0.9354	20.7
DNTD (with spectra shifting)	99.9	3.6	0.9738	13.2	0.9990	2.6	0.9993	2.2
DNTD (with FIA profiles shifting)	67.0	32.9	**0.6521**	**49.2**	**0.8706**	**29.4**	0.9972	4.3
MCR-ALS bilinear (0,0,0)	97.0	17.4	**0.8614**	**30.5**	**0.8391**	**33.0**	0.9766	12.4
MCR-ALS trilinear (1,1,1)	96.9	17.7	**0.8683**	**29.7**	**0.8138**	**35.5**	0.9865	9.4
MCR-ALS trilinear (2,2,2)	96.3	19.2	**0.8733**	**29.1**	**0.8265**	**34.3**	0.9853	9.8

Supplementary Figures

- Figures S1-S8: ATLD, DNTD, PARAFAC, PARAFAC2 and MCR-ALS (different variants) results in the analysis of the wine GC-MS dataset
- Figures: S9-S14 ATLD, DNTD, PARAFAC and PARAFAC2 and MCR-ALS (different variants) results in the analysis of the FIA dataset using 6 components
- Figures: S15-S21 ATLD, DNTD, PARAFAC and PARAFAC2 and MCR-ALS (different variants) results in the analysis of the FIA dataset using 3 components

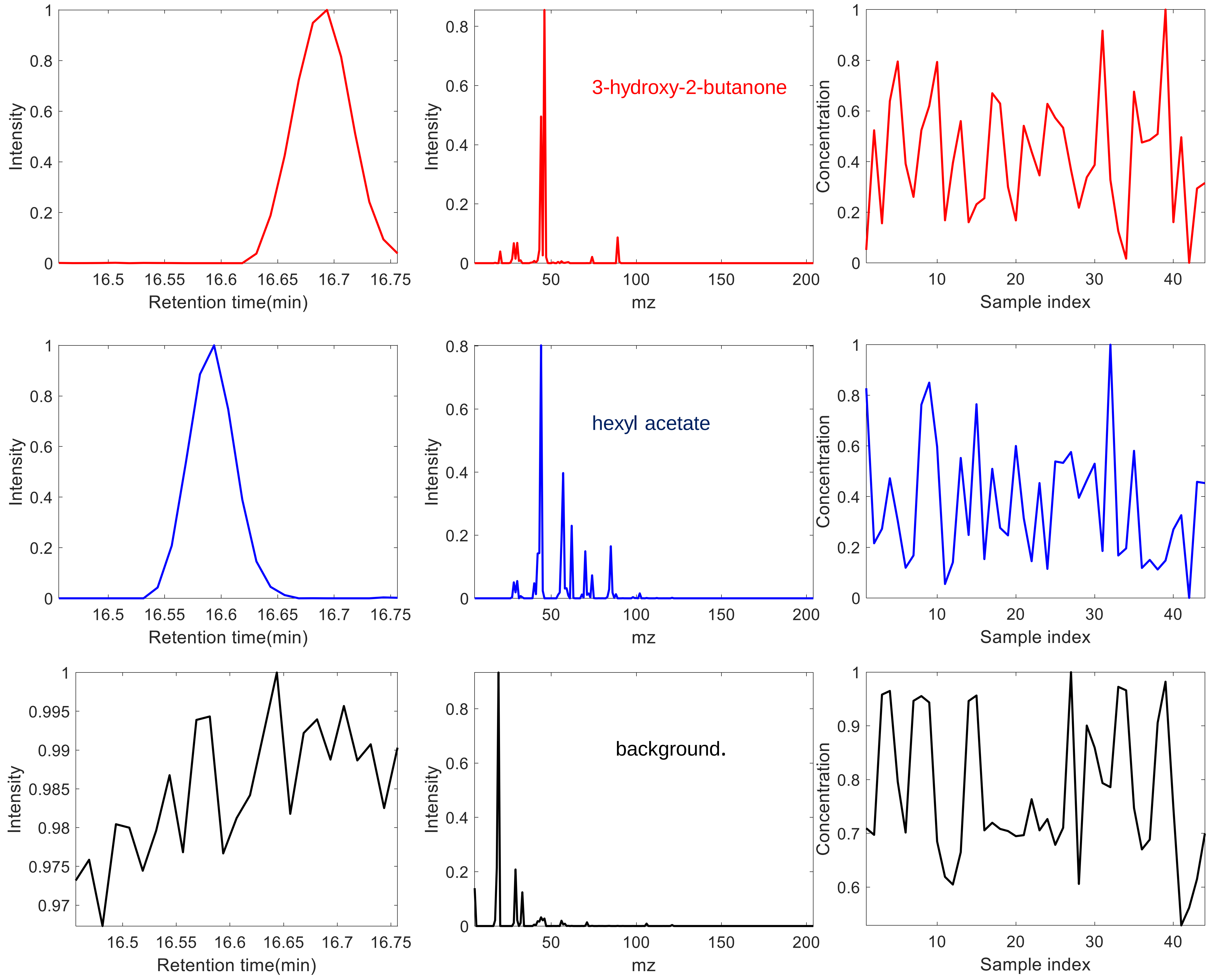

Figure S1 Elution, spectra, and sample profiles of the three components resolved by **ATLD** in the analysis of the wine GC-MS dataset.

Figure S1

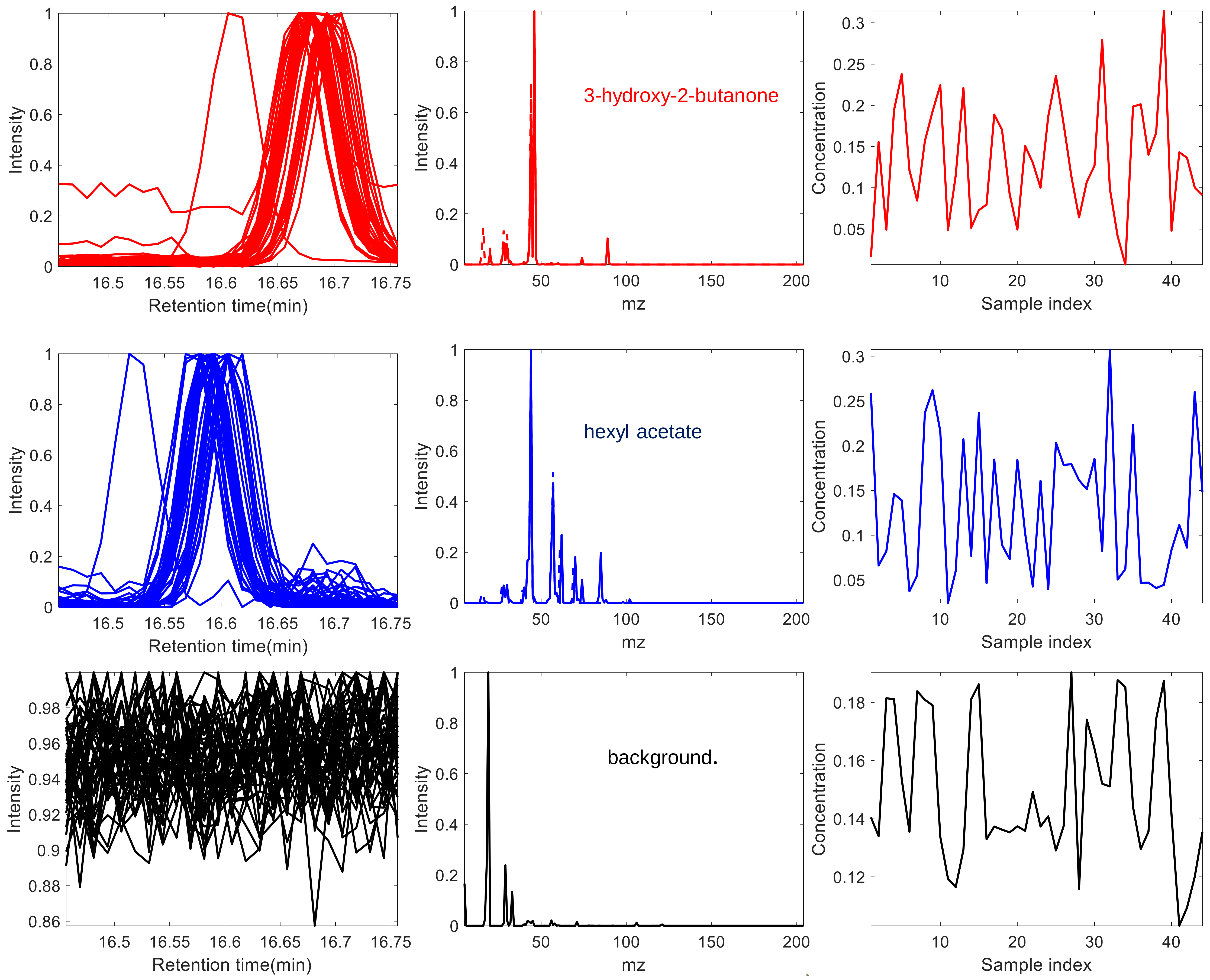

Figure S2 Elution, spectra, and sample profiles of the three components resolved by **DNTD** in the analysis of the wine GC-MS dataset.

Figure S2

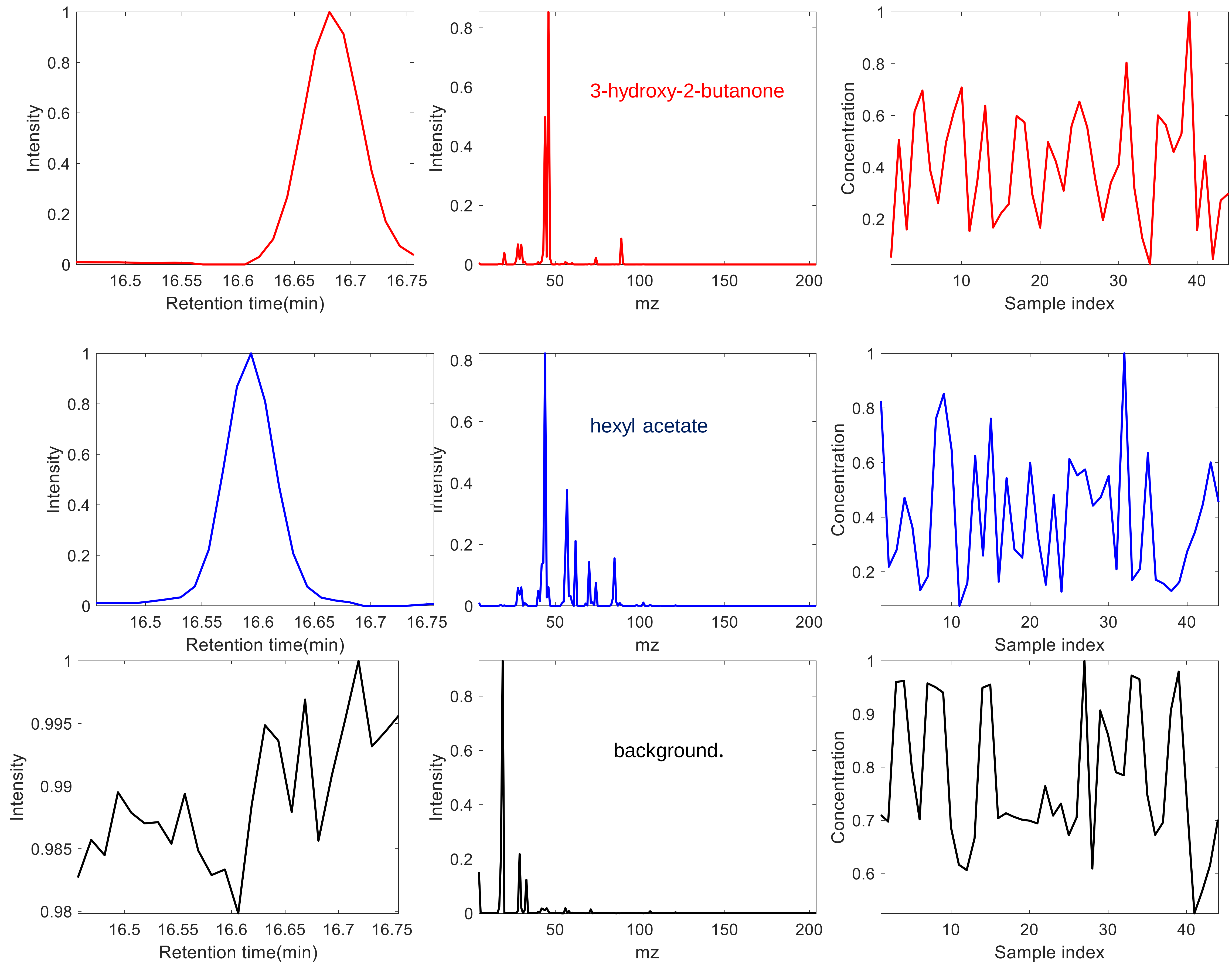

Figure S3 Elution, spectra, and sample profiles of the three components resolved by **PARAFAC** in the analysis of the wine GC-MS dataset.

Figure S3

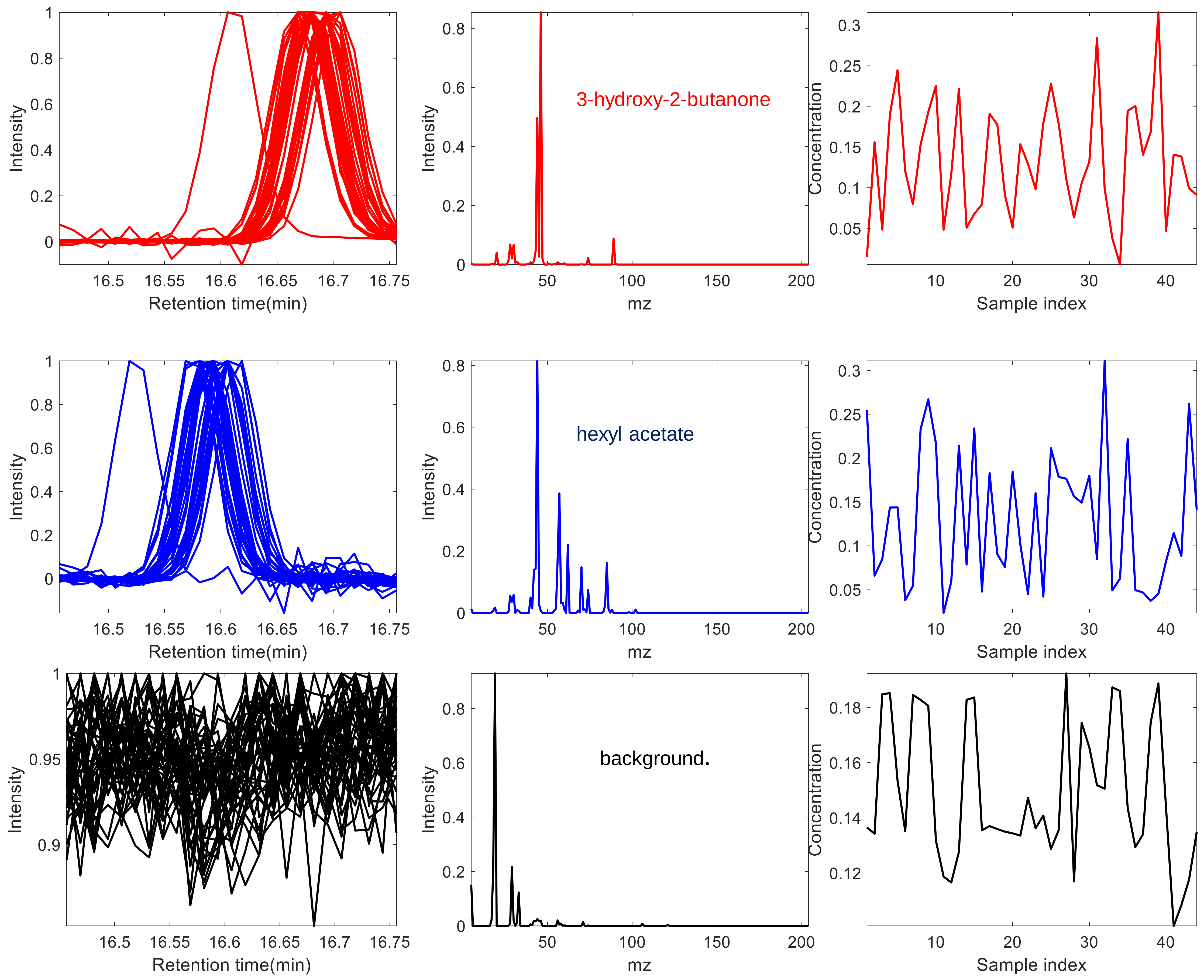

Figure S4 Elution, spectra, and sample profiles of the three components resolved by **PARAFAC2** in the analysis of the wine GC-MS dataset.

Figure S4

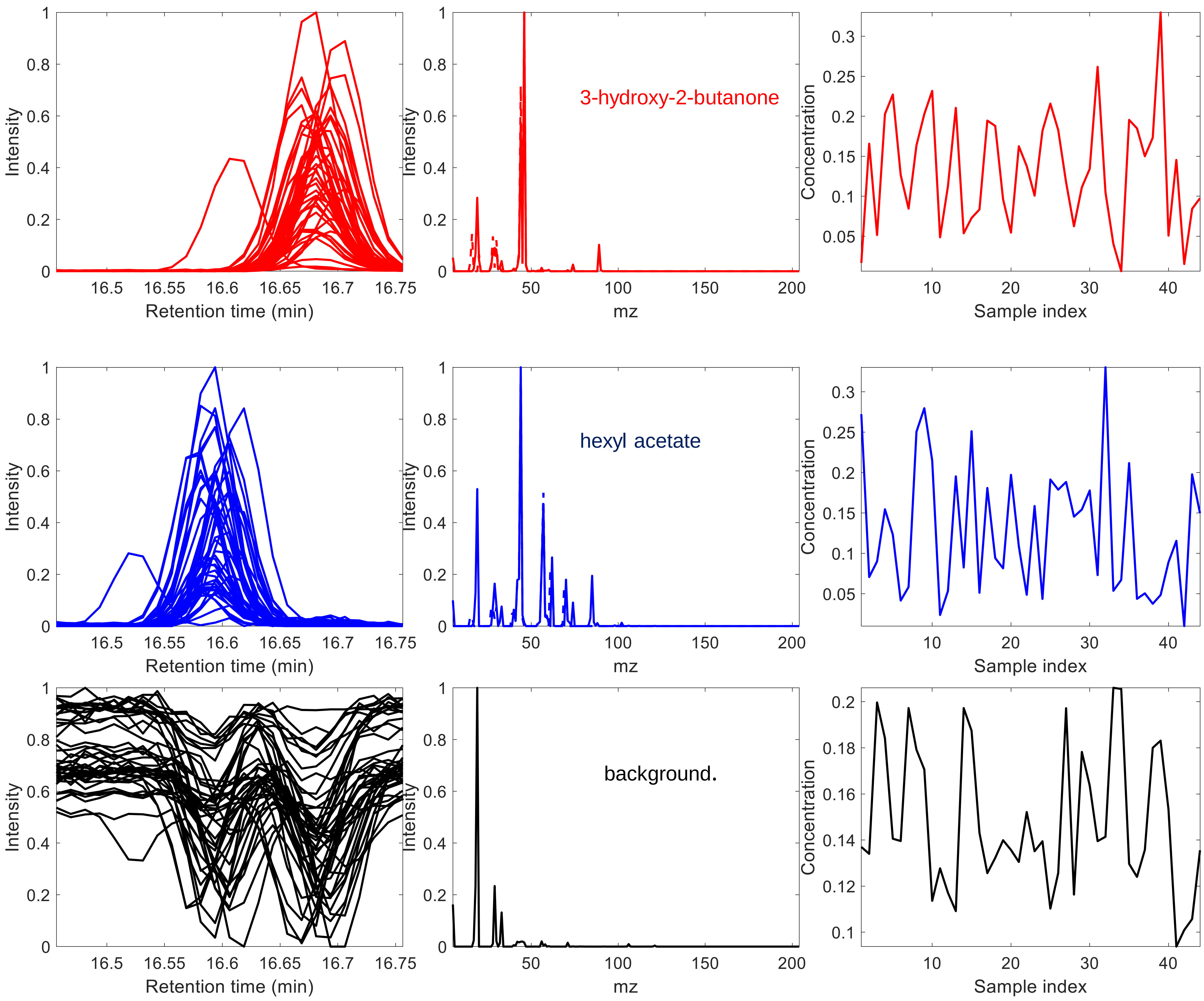

Figure S5 Elution, spectra, and sample profiles of the three components resolved by **MCR-ALS bilinear (0,0,0)** in the analysis of the wine GC-MS dataset.

Figure S5

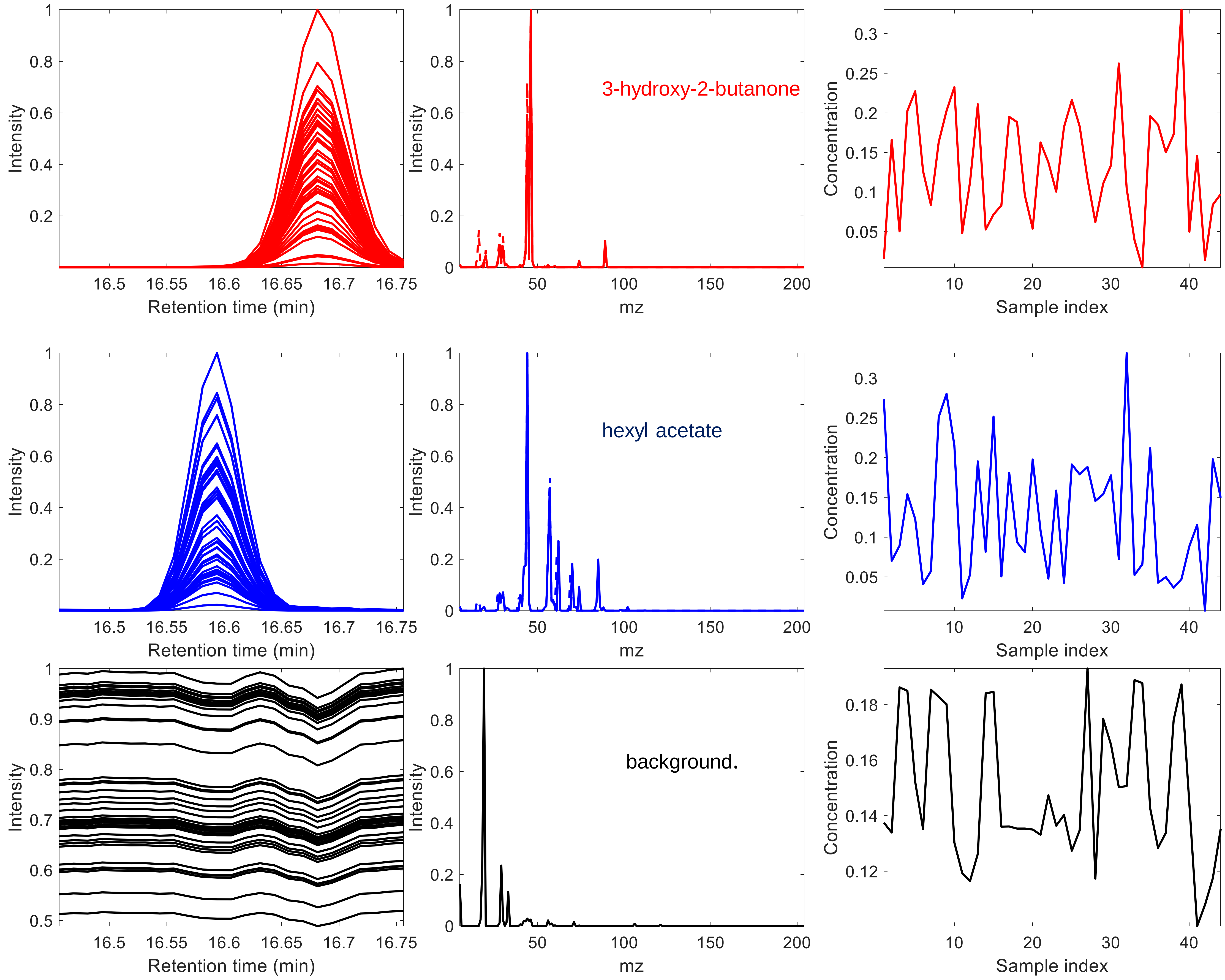

Figure S6 Elution, spectra, and sample profiles of the three components resolved by **MCR-ALS trilinear (1,1,1)** in the analysis of the wine GC-MS dataset.

Figure S6

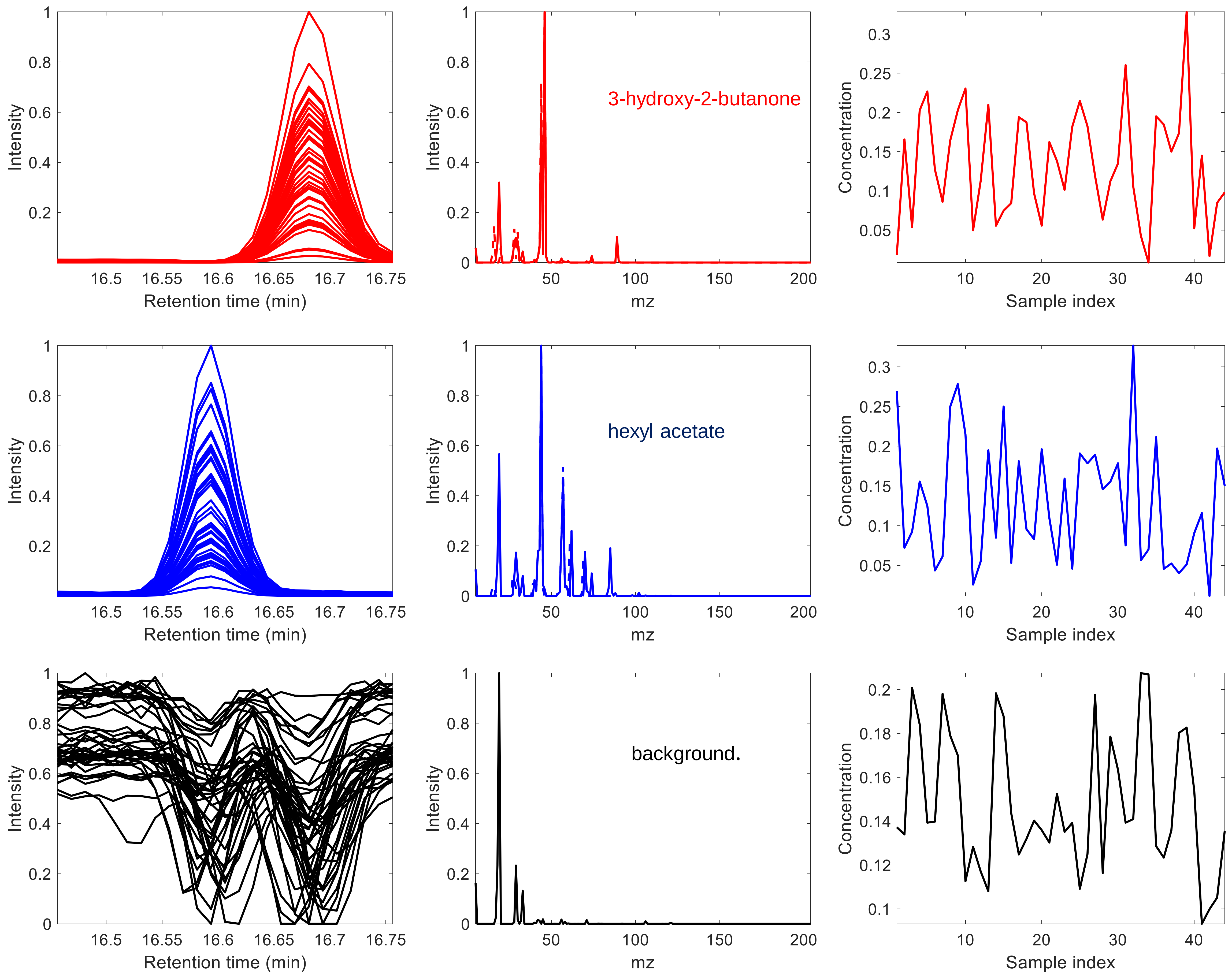

Figure S7 Elution, spectra, and sample profiles of the three components resolved by **MCR-ALS trilinear (1,1,0)** in the analysis of the wine GC-MS dataset.

Figure S7

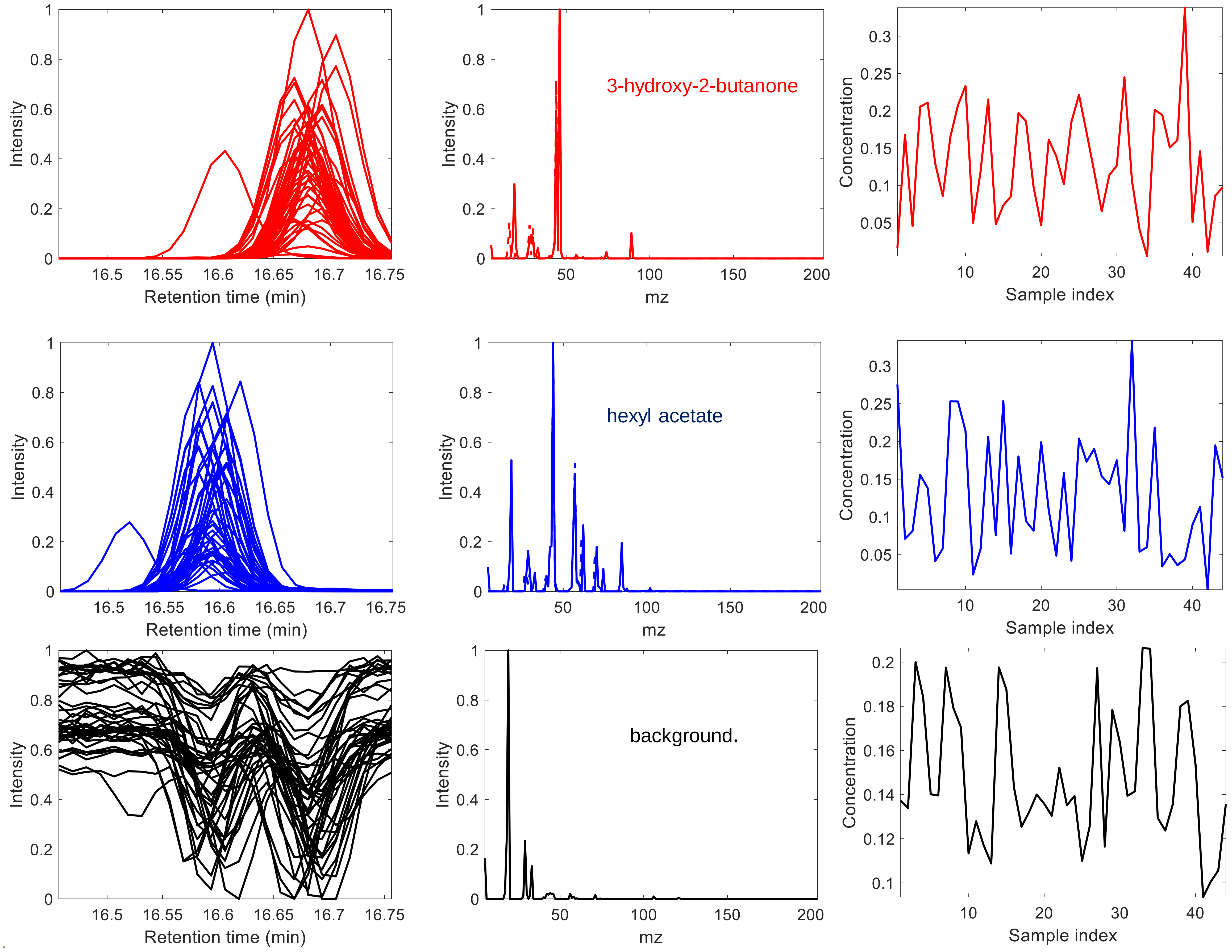

Figure S8 Elution, spectra, and sample profiles of the three components resolved by **MCR-ALS trilinear (2,2,0)** in the analysis of the wine GC-MS dataset.

Figure S8

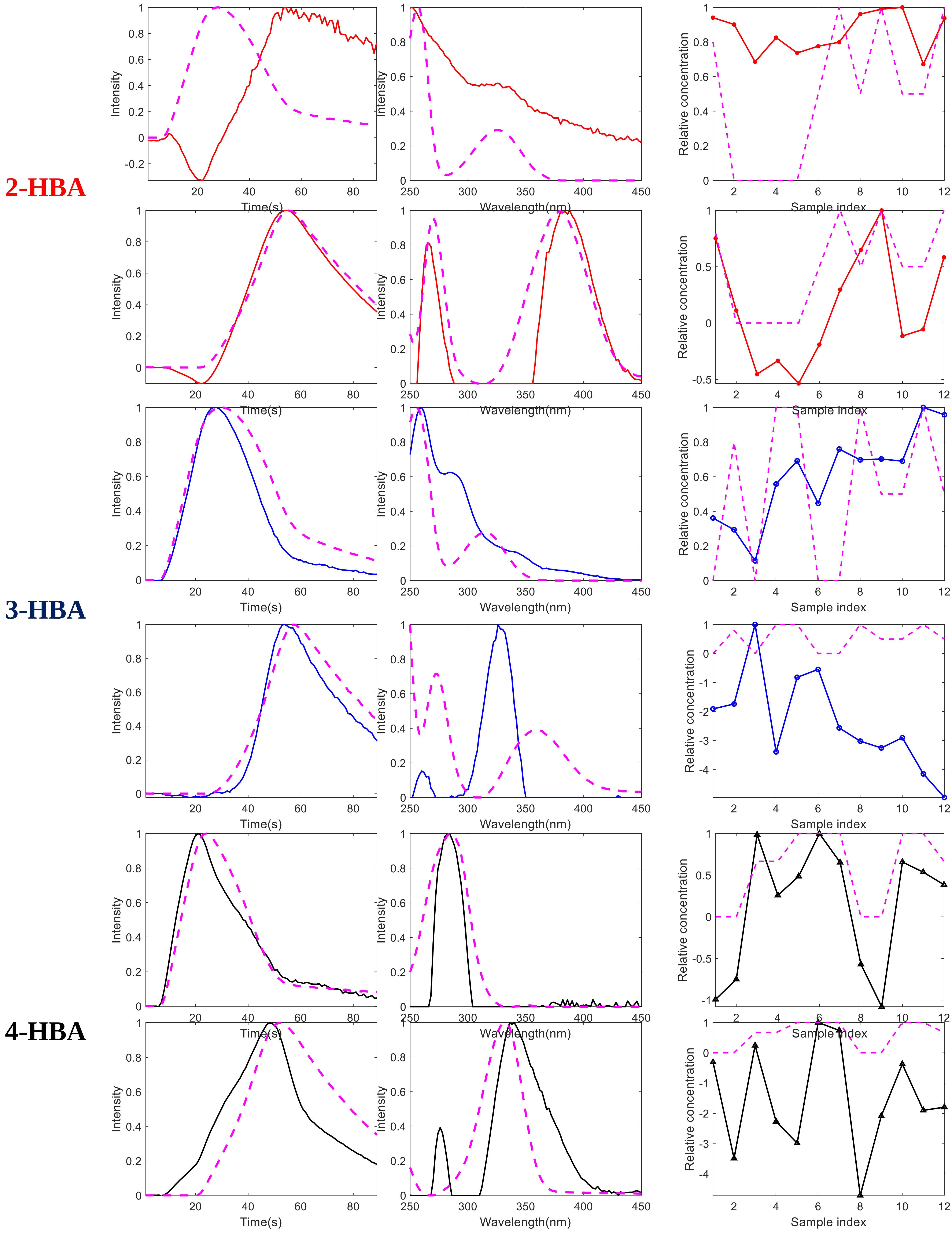

Figure S9 FIA (left), spectra (middle), and sample (right) profiles of the six components resolved in the analysis of the pH gradient FIA-UV dataset by **ATLD,** corresponding to the two acid-base species of 2-HBA (red), 3-HBA (blue) and 4-HBA (black). Species spectra and sample profiles were compared with reference ones (cyan). See results in section 4.3 and Table 3.

Figure S9

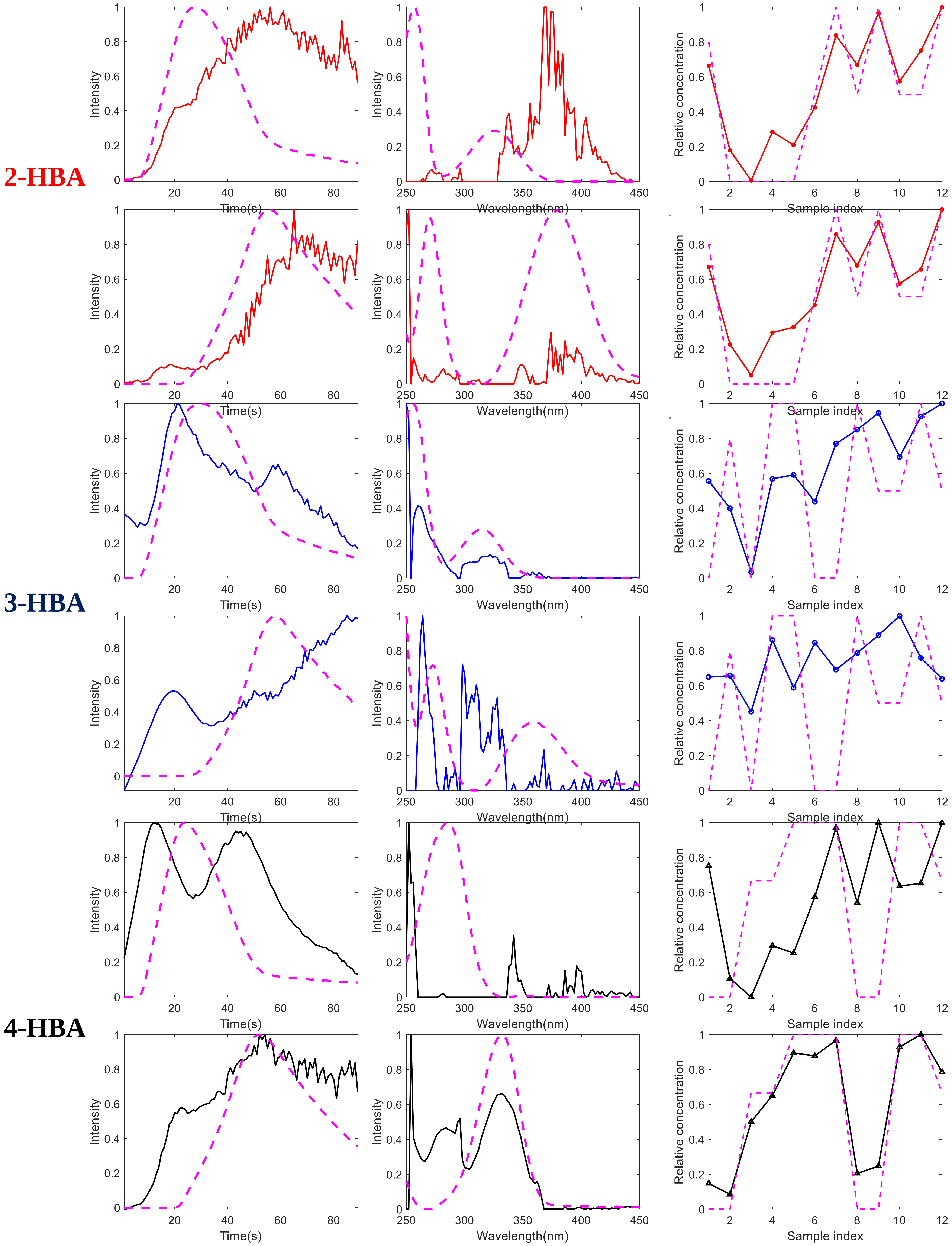

Figure S10 FIA (left), spectra (middle), and sample (right) profiles of the six components resolved in the analysis of the pH gradient FIA-UV dataset by **DNTD**, corresponding to the two acid-base species of 2-HBA (red), 3-HBA (blue) and 4-HBA (black). Species spectra and sample profiles were compared with reference ones (cyan). See results in section 4.3 and Table 3.

Figure S10

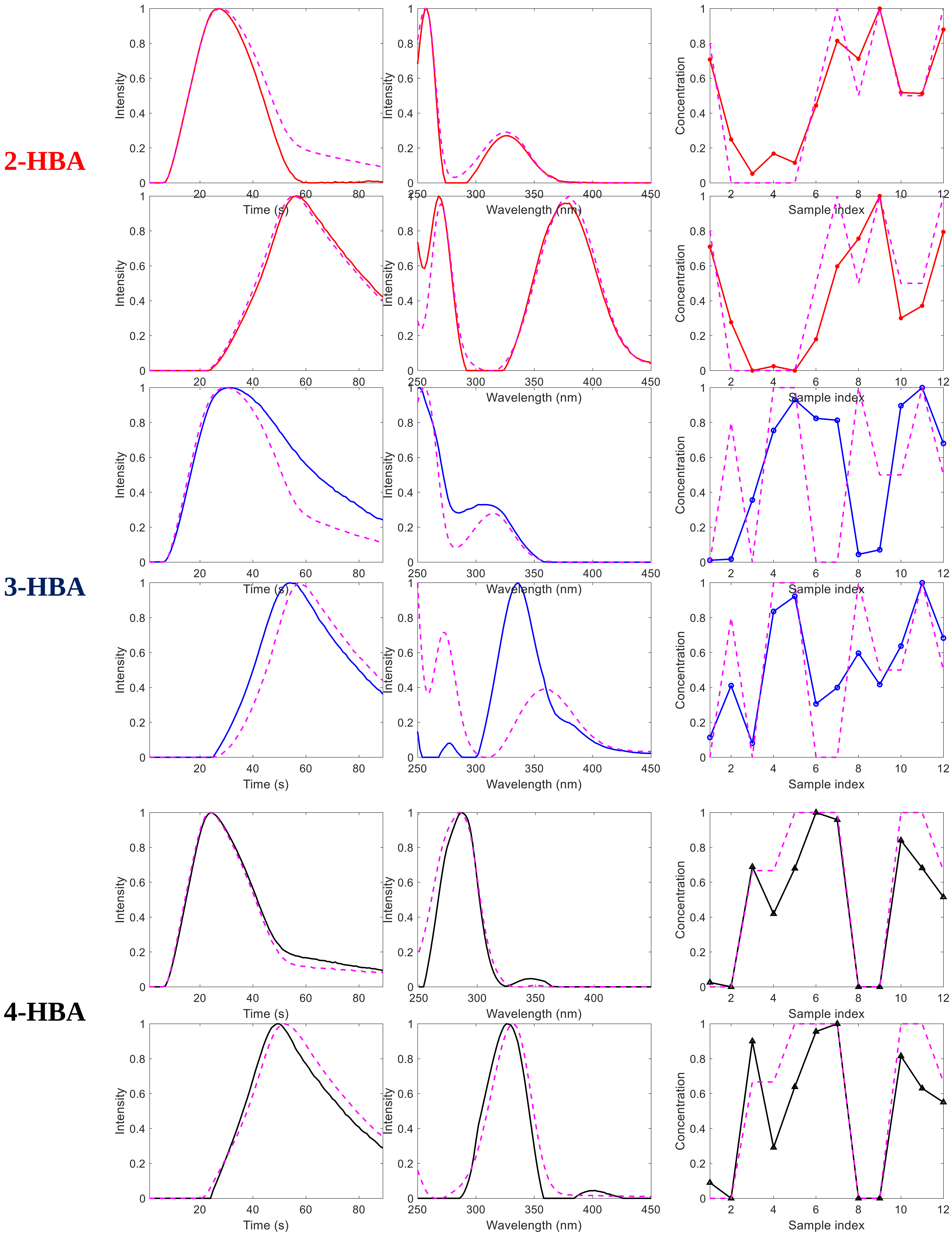

Figure S11 FIA (left), spectra (middle), and sample (right) profiles of the six components resolved in the analysis of the pH gradient FIA-UV dataset by **PARAFAC**, corresponding to the two acid-base species of 2-HBA (red), 3-HBA (blue) and 4-HBA (black). Species spectra and sample profiles were compared with reference ones (cyan). See results in section 4.3 and Table 3.

Figure S11

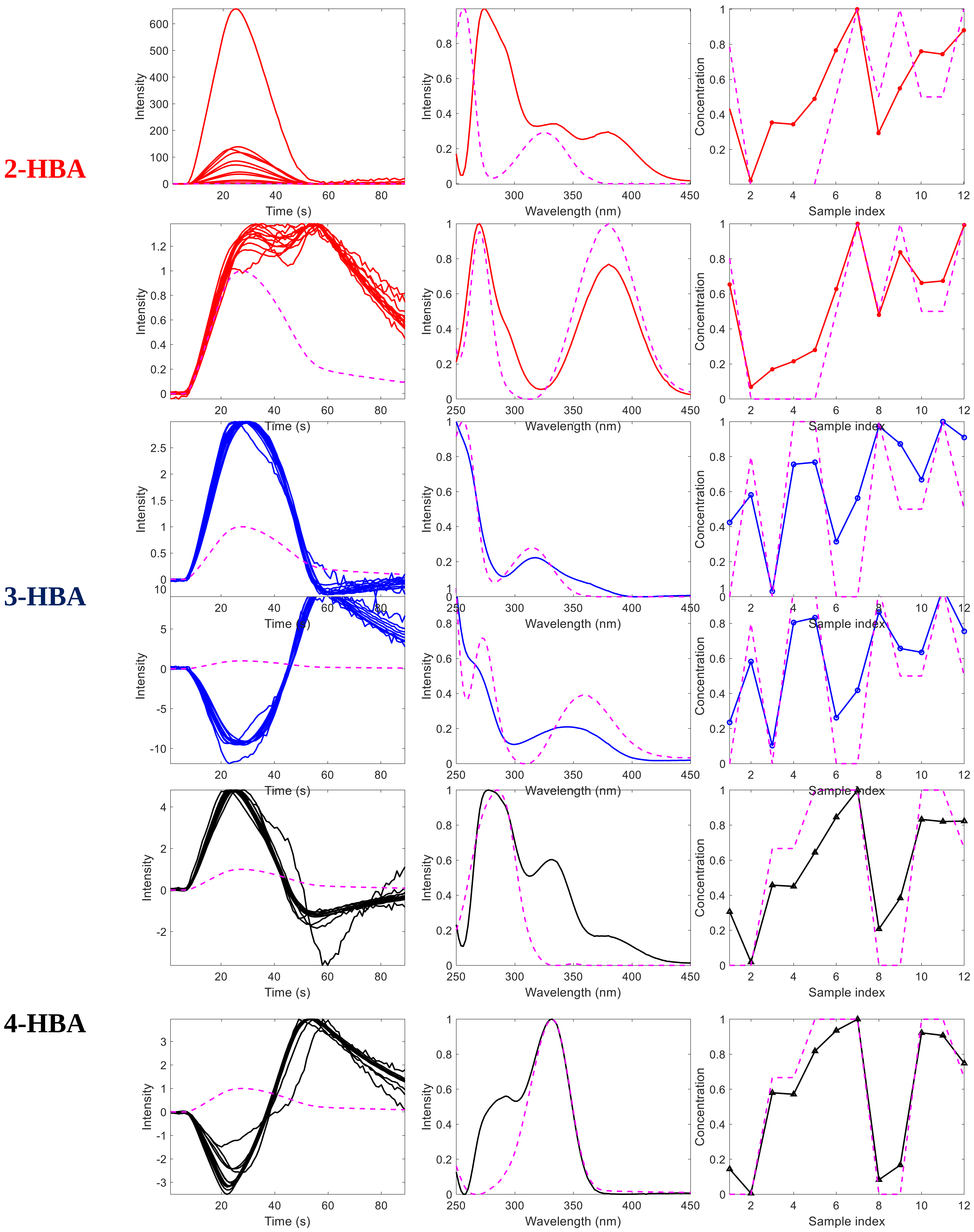

Figure S12 FIA (left), spectra (middle), and sample (right) profiles of the six components resolved in the analysis of the pH gradient FIA-UV dataset by **PARAFAC2**, corresponding to the two acid-base species of 2-HBA (red), 3-HBA (blue) and 4-HBA (black). Species spectra and sample profiles were compared with reference ones (cyan). See results in section 4.3 and Table 3.

Figure S12

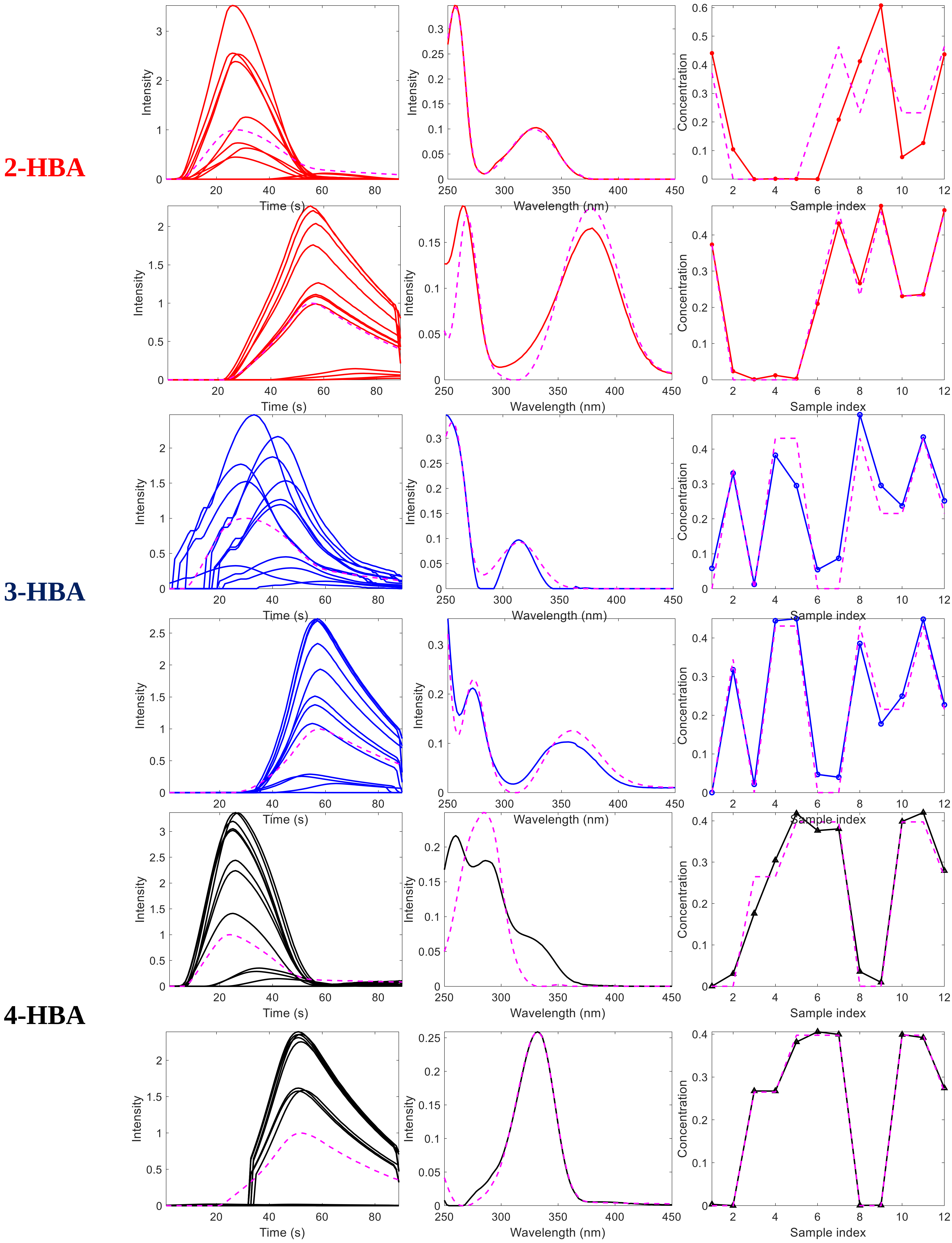

Figure S13 FIA (left), spectra (middle), and sample (right) profiles of the six components resolved in the analysis of the pH gradient FIA-UV dataset by **MCR-ALS trilinear (1,1,1)** corresponding to the two acid-base species of 2-HBA (red), 3-HBA (blue) and 4-HBA (black). Species spectra and sample profiles were compared with reference ones (cyan). See results in section 4.3 and Table 3.

Figure S13

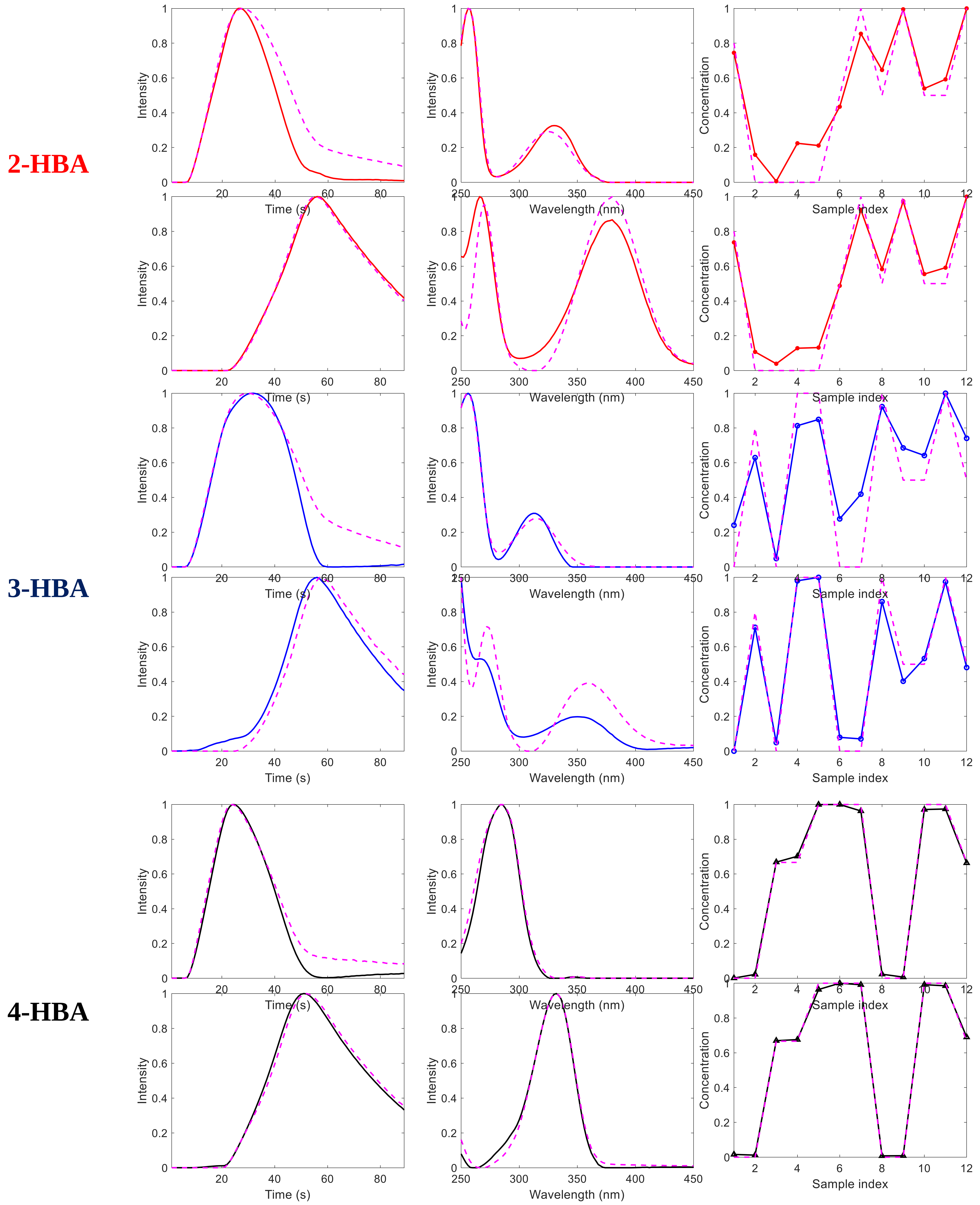

Figure S14 FIA (left), spectra (middle), and sample (right) profiles of the six components resolved in the analysis of the pH gradient FIA-UV dataset by **MCR-ALS trilinear (2,2,2)** corresponding to the two acid-base species of 2-HBA (red), 3-HBA (blue) and 4-HBA (black). Species spectra and sample profiles were compared with reference ones (cyan). See results in section 4.3 and Table 3.

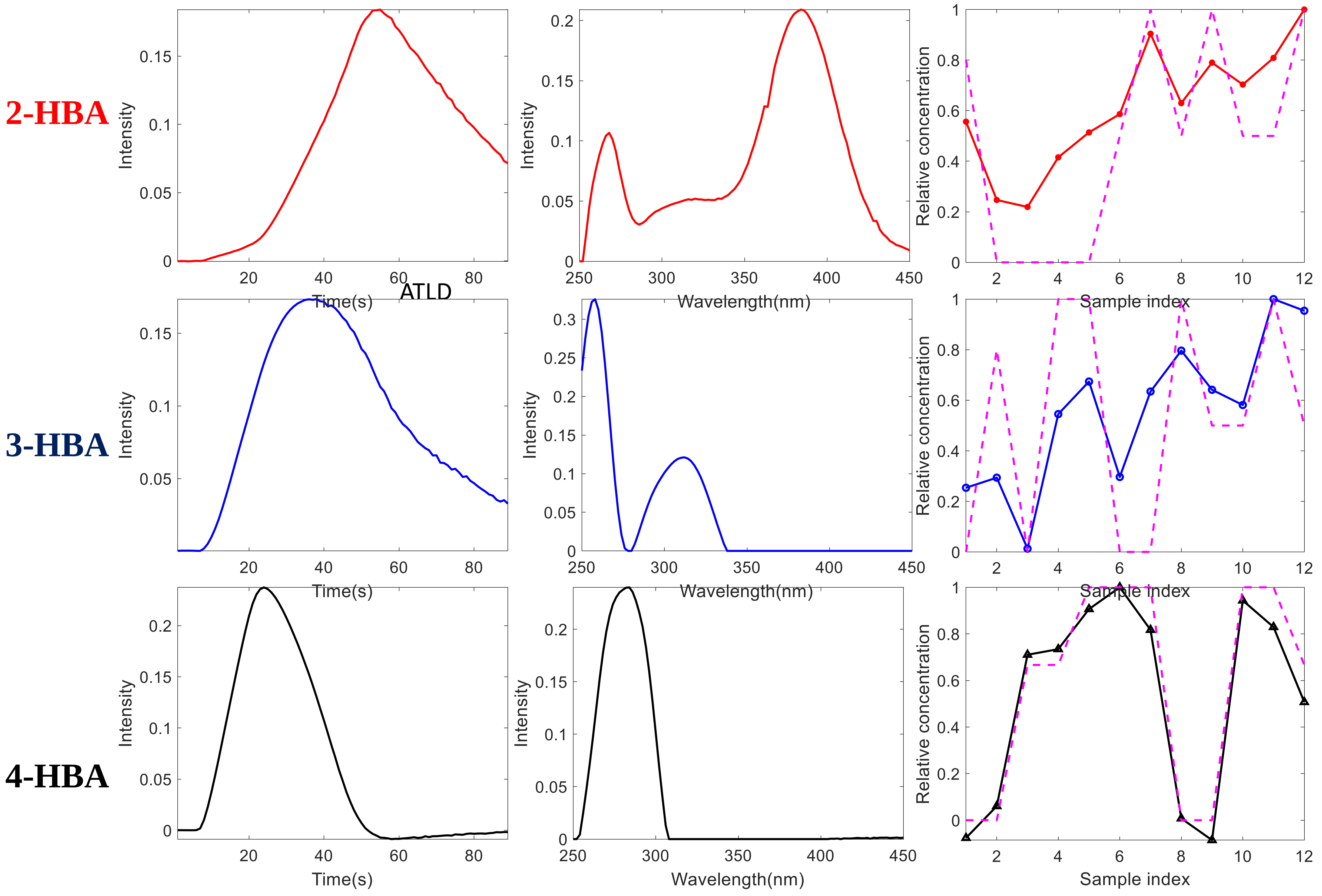

Figure S15 FIA (left), spectra (middle), and sample (right) profiles using three components resolved in the analysis of the pH gradient FIA-UV dataset by **ATLD** of 2-HBA (red), 3-HBA (blue) and 4-HBA (black). Sample profiles were compared with reference ones (cyan). See results in section 4.3 and Table 3.

Figure S15

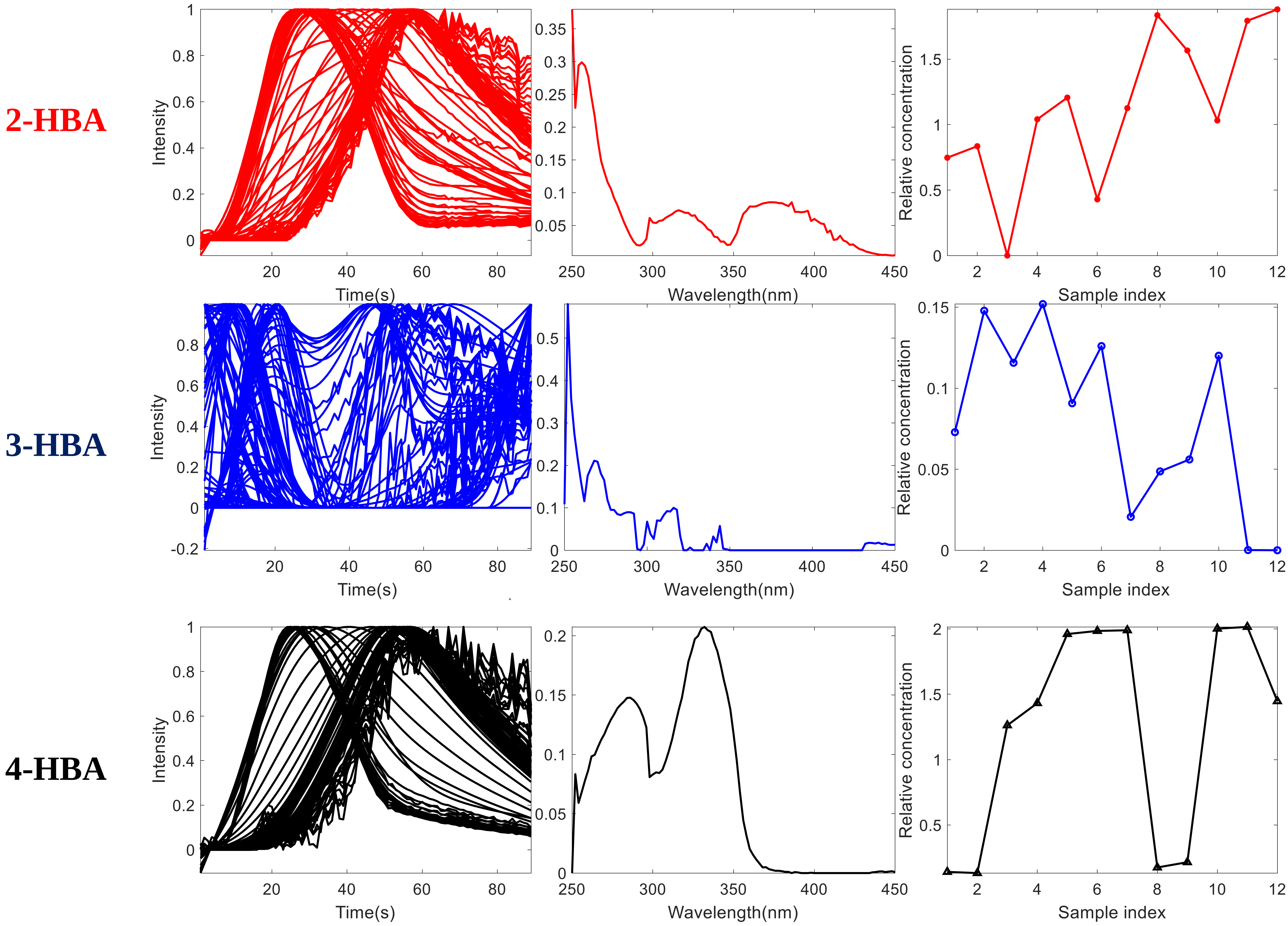

Figure S16 FIA (left), spectra (middle), and sample (right) profiles using three components resolved in the analysis of the pH gradient FIA-UV dataset by **DNTD** of 2-HBA (red), 3-HBA (blue) and 4-HBA (black). Sample profiles were compared with reference ones (cyan). See results in section 4.3 and Table 3.

Figure S16

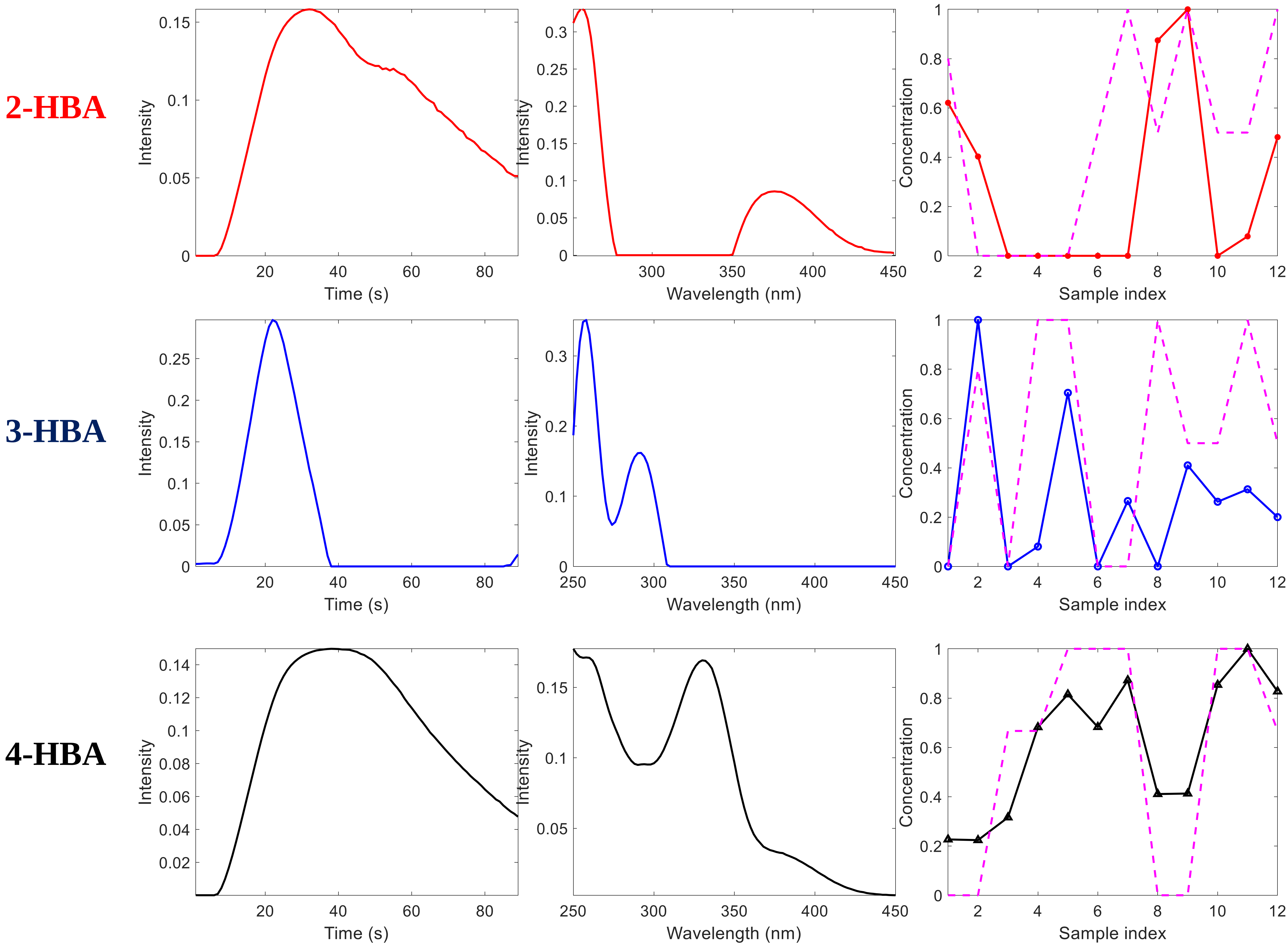

Figure S17 FIA (left), spectra (middle), and sample (right) profiles using three components resolved in the analysis of the pH gradient FIA-UV dataset by **PARAFAC** of 2-HBA (red), 3-HBA (blue) and 4-HBA (black). Sample profiles were compared with reference ones (cyan). See results in section 4.3 and Table 3.

Figure S17

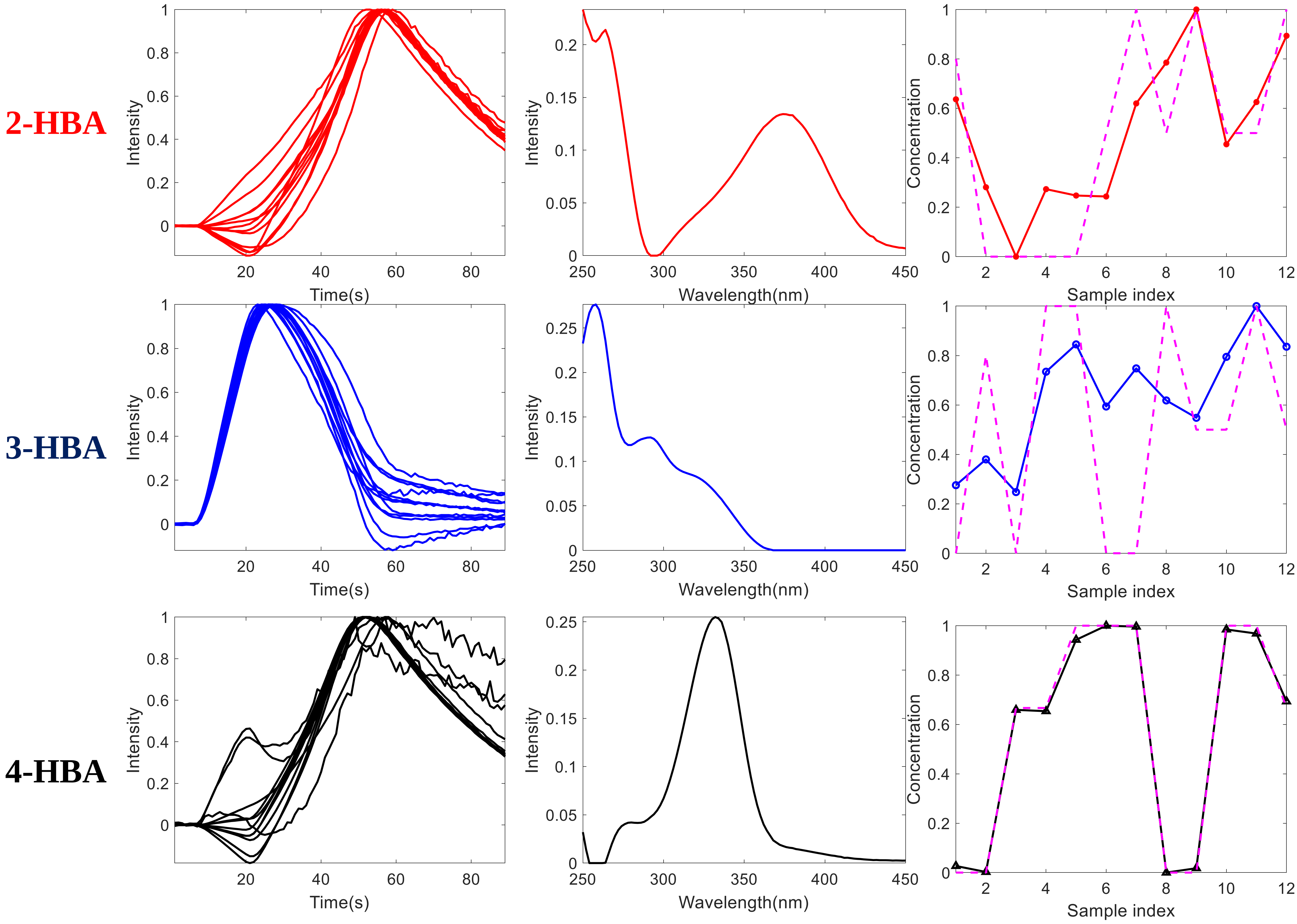

Figure S18 FIA (left), spectra (middle), and sample (right) profiles using three components resolved in the analysis of the pH gradient FIA-UV dataset by **PARAFAC2** of 2-HBA (red), 3-HBA (blue) and 4-HBA (black). Sample profiles were compared with reference ones (cyan). See results in section 4.3 and Table 3.

Figure S18

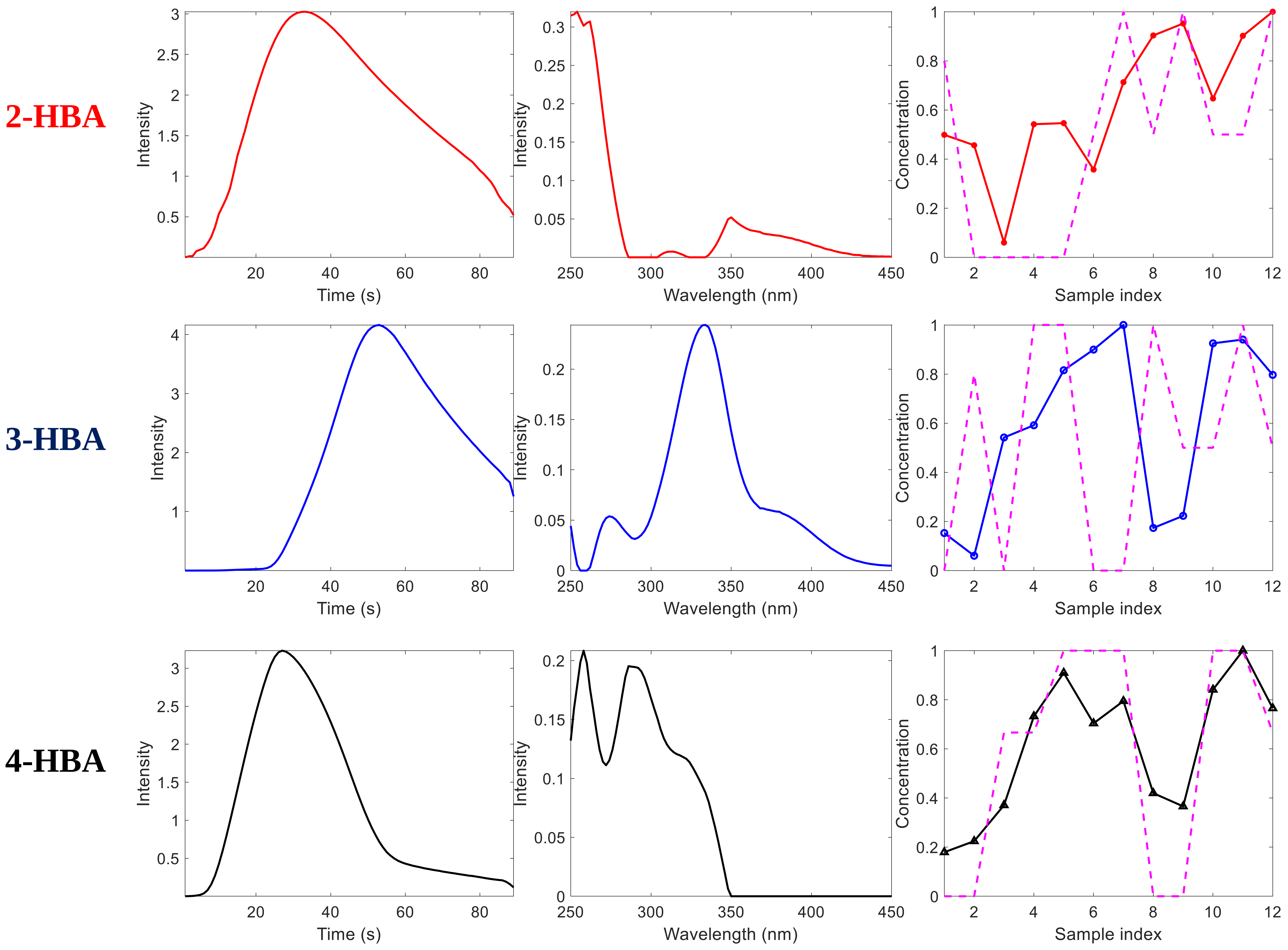

Figure S19 FIA (left), spectra (middle), and sample (right) profiles using three components resolved in the analysis of the pH gradient FIA-UV dataset by **MCR-ALS trilinear (1,1,1)** of 2-HBA (red), 3-HBA (blue) and 4-HBA (black). Sample profiles were compared with reference ones (cyan). See results in section 4.3 and Table 3.

Figure S19

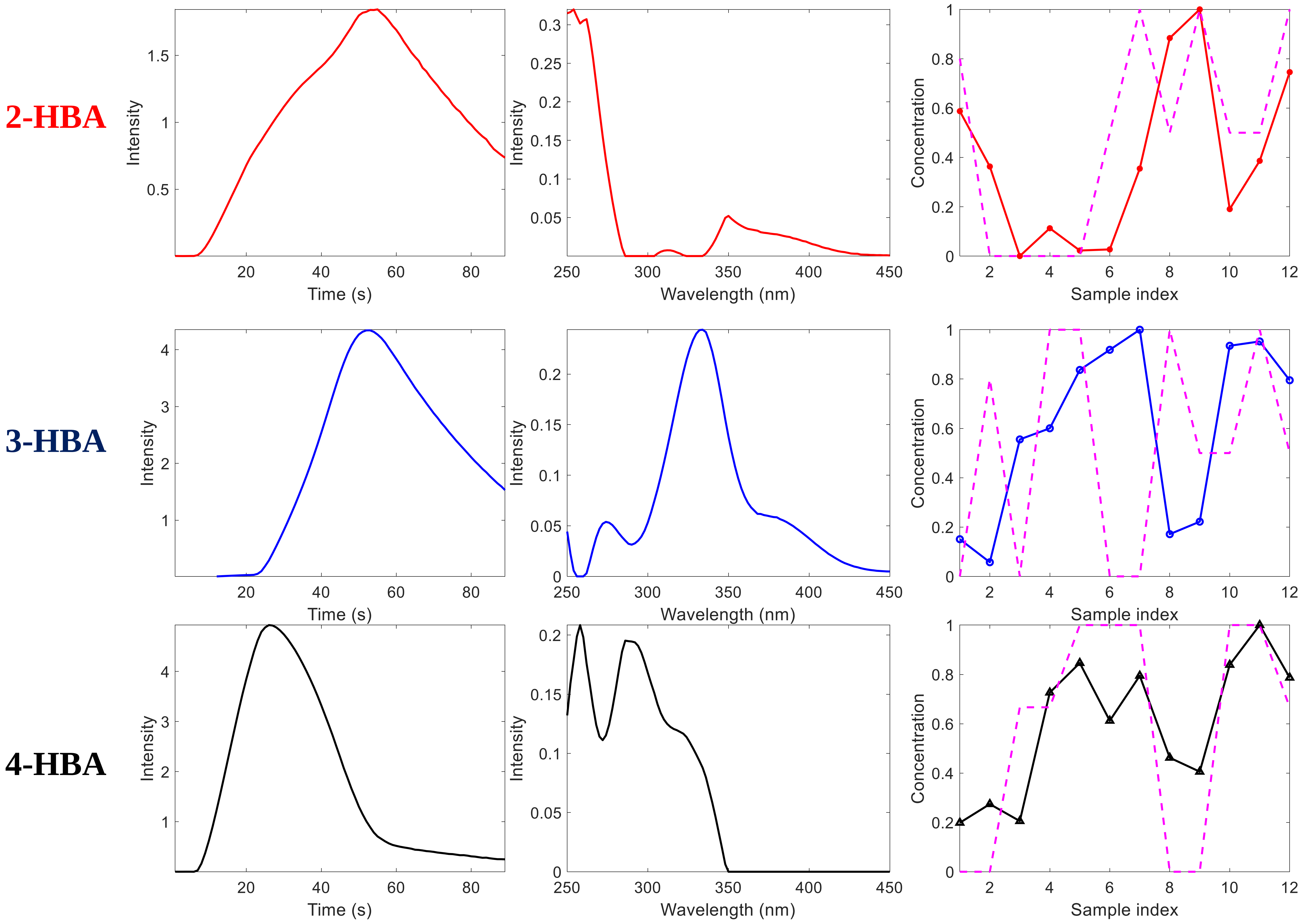

Figure S20 FIA (left), spectra (middle), and sample (right) profiles using three components resolved in the analysis of the pH gradient FIA-UV dataset by **MCR-ALS trilinear (2,2,2)** of 2-HBA (red), 3-HBA (blue) and 4-HBA (black). Sample profiles were compared with reference ones (cyan). See results in section 4.3 and Table 3.

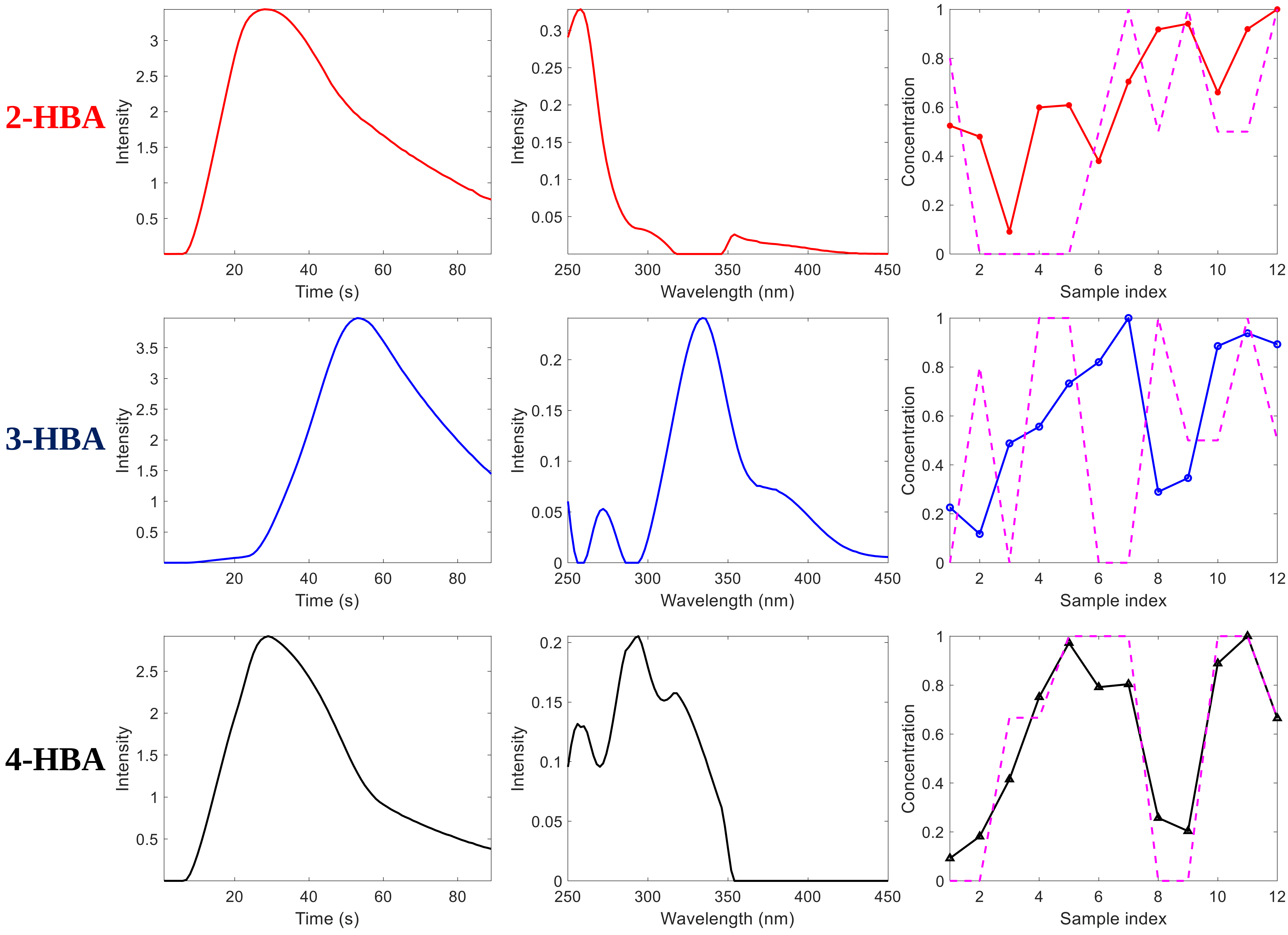

Figure S21 FIA (left), spectra (middle), and sample (right) profiles using three components resolved in the analysis of the pH gradient FIA-UV dataset by **MCR-ALS bilinear (0,0,0)** of 2-HBA (red), 3-HBA (blue) and 4-HBA (black). Sample profiles were compared with reference ones (cyan). See results in section 4.3 and Table 3.

Figure S21
